# Ultrasonographic assessment of optic nerve sheath diameter on the affected and unaffected sides as a predictor of clinical deterioration at discharge in patients with large hemispheric infarction

**DOI:** 10.3389/fneur.2025.1644281

**Published:** 2025-10-15

**Authors:** Changlan Li, Yanping Li, Xunhu Gu, Yancai Zhang, Xiaoqian Cheng, Wei Wang

**Affiliations:** Department of Neurology, The Second Affiliated Hospital, Jiangxi Medical College, Nanchang University, Nanchang, China

**Keywords:** large hemispheric infarction, intracranial pressure, ultrasound, optic nerve sheath diameter, prognosis

## Abstract

**Background and objectives:**

Progressive cerebral edema worsens prognosis in large hemispheric infarction (LHI). Ultrasonographic optic nerve sheath diameter (ONSD) measurement provides a non-invasive method for estimating intracranial pressure. This study aimed to evaluate the predictive value of affected- and unaffected-side ONSD for clinical deterioration at discharge in LHI patients and to provide reference data on factors linked to deterioration, follow-up outcomes, and longitudinal ONSD monitoring.

**Methods:**

This retrospective study enrolled 35 LHI patients, classified into two groups: improved and deteriorated, based on clinical and imaging findings at discharge. Demographic and clinical characteristics were compared. Receiver operating characteristic (ROC) analysis evaluated the predictive performance of affected- and unaffected-side ONSD. Area under the curve (AUC) difference was compared using the DeLong test. Post-discharge follow-up at 30 and 90 days was conducted via telephone interviews. Longitudinal changes in affected- and unaffected-side ONSD were depicted using line graphs for patients with extended stays in the neuro-intensive care unit (NICU).

**Results:**

Statistically significant variables included age (*p* = 0.002), female (*p* = 0.002), history of atrial fibrillation (*p* = 0.044), GCS score (*p* = 0.028), affected-side ONSD (*p* = 0.002), unaffected-side ONSD (*p* = 0.012), and duration of NICU stay (*p* = 0.002). A positive linear correlation was identified between ONSD values and discharge outcomes. The optimal cut-off for predicting deterioration was 5.54 mm for affected-side ONSD (sensitivity 81.3%, specificity 78.9%, AUC = 0.814) versus 5.57 mm for the unaffected-side (68.8, 78.9%, AUC = 0.757), with no significant AUC difference between sides. The overall 30-day post-discharge mortality was 39.29%. Longitudinal changes in bilateral ONSD showed remarkable overlap in both individual and group observations. Patients with improved outcomes exhibited decreasing ONSD trends, whereas those with deterioration displayed increasing trends.

**Conclusion:**

Preoperative ONSD measurement is a feasible and practical predictor of discharge prognosis in LHI patients, with both affected and unaffected sides providing reliable monitoring. Factors including age, female, history of atrial fibrillation, GCS score, ONSD on either side, and NICU stay duration may influence outcomes. The high short-term mortality underscores the importance of the post-discharge transition management. The clinical value of longitudinal ONSD monitoring requires further investigation.

## 1 Introduction

Large hemispheric infarction (LHI), most commonly caused by proximal middle cerebral artery (MCA) occlusion, accounts for approximately 2 to 8% of all hemispheric cerebral infarctions (1), with an annual incidence ranging from 10 to 20 per 100,000 individuals (2). Mortality rates remain alarmingly high, reaching up to 80%, and nearly two-thirds of survivors experience significant disability (3). A major contributor to this poor prognosis is malignant cerebral edema (MCE). Early decompressive hemicraniectomy (DHC) has been shown to effectively reduce the mass effect caused by edema, thereby decreasing mortality and improving functional outcomes (1). However, most studies have focused on the first 48 h after symptom onset, leaving a significant evidence gap regarding the benefits and optimal timing of DHC beyond this period. Given the procedural risks, substantial costs, and reliance on specialized neurosurgical resources, clinical management of LHI varies widely. Therefore, preventing the progression of MCE is critical, underscoring the urgent need for early intracranial pressure (ICP) monitoring and timely initiation of therapies aimed at controlling ICP and limiting edema expansion.

Invasive techniques, such as intraventricular monitoring, remain the gold standard for ICP assessment. However, these procedures carry inherent risks, including hemorrhage and infection (4). Current expert consensus advises against routine invasive ICP monitoring in LHI patients not undergoing DHC (5). Ultrasonographic measurement of the optic nerve sheath diameter (ONSD) has demonstrated a strong correlation with ICP (6, 7), showing an approximately linear relationship across a wide pressure range (8), which supports its use as a non-invasive alternative for ICP evaluation.

To date, studies on ONSD in LHI populations remain limited (9, 10), and most have focused on measurements taken within 24 h of symptom onset. Some lacked comprehensive documentation of treatments administered before or after ONSD assessment. Importantly, post-DHC ONSD measurements may not accurately reflect true ICP values (11). To better reflect real-world clinical scenarios, where patients often receive initial treatment before admission or experience delays in transfer, we extended the ONSD measurement window to within 48 h after symptom onset. Given the documented synchronous changes between the left and right eyes in stroke patients (12), earlier investigations primarily used the mean bilateral ONSD as the primary metric. However, whether the side of infarction affects ONSD values remains unclear. Therefore, this study aims to evaluate the predictive value of ONSD measurements from the affected and unaffected sides for clinical deterioration at discharge in LHI patients who received initial treatment while retaining an intact cranium. Our findings may provide valuable insights for early ICP assessment and prognostic evaluation in this population. Furthermore, we aim to provide reference data for future research designs by identifying factors associated with clinical deterioration, evaluating functional outcomes over the follow-up period, and characterizing the longitudinal changes in ONSD during treatment.

## 2 Materials and methods

### 2.1 Patient selection

This study was approved by the Medical Ethics Committee of the Second Affiliated Hospital of Nanchang University (Reference Code: MR-36-25-024684), with a waiver of informed consent.

Patients were admitted to the neurology emergency department of the Second Affiliated Hospital of Nanchang University. Subsequently, they transferred to the neuro-intensive care unit (NICU) for treatment between November 2023 and October 2024 and were enrolled retrospectively.

Inclusion criteria were as follows: (1) clinical presentation consistent with anterior circulation infarction; (2) imaging diagnosis based on established guidelines: non-contrast computed tomography (CT) within 6 h of symptom onset showing infarction involving >1/3 of the MCA territory, or non-contrast CT within 6 to 7 days post-onset demonstrating infarction > 1/2 of the MCA territory; alternatively, diffusion-weighted imaging (DWI) within 6 h after onset with infarct volume > 80 mL, or DWI within 14 h post-onset with infarct volume > 145 mL (13); (3) age ≥ 18 years; (4) completion of imaging, laboratory tests, ONSD ultrasound measurements on both the affected and unaffected sides, Glasgow Coma Scale (GCS), and National Institutes of Health Stroke Scale (NIHSS) assessments within 48 h after symptom onset.

Exclusion criteria were as follows: (1) age < 18 years; (2) time from symptom onset > 48 h; (3) bilateral hemispheric infarction; (4) imaging evidence of hemorrhagic transformation, cerebral hemorrhage, subarachnoid hemorrhage, aneurysm, arteriovenous malformation, space-occupying lesion, or other conditions potentially affecting ICP; (5) known history of ocular and optic nerve disorders, such as glaucoma, cataract or optic neuritis; (6) presence of skull fracture with cerebrospinal fluid leakage; (7) inability to cooperate with ultrasonographic examination; (8) in-hospital mortality.

Finally, a total of 35 patients diagnosed with LHI were included in the study. All participants were managed in accordance with standardized protocols for the stroke unit. Treatments were administered after obtaining informed consent from the patients or their legal surrogates.

Deterioration at discharge was defined as clinical worsening due to progressive cerebral edema, after excluding other causes of neurological decline (e.g., metabolic disturbances). Patients meeting any of the following criteria were classified into the deterioration group (Group 2); those who did not meet these criteria were assigned to the improved group (Group 1): 1. progressive impairment of consciousness, reflected by an increase of ≥1 point in the NIHSS consciousness item (e.g., from 1 to 2), or new-onset pupillary asymmetry; 2. midline shift ≥5 mm on follow-up CT compared to baseline imaging (14).

### 2.2 Demographic and clinical characteristics

Demographic and clinical data were collected from the hospital’s electronic medical records by two neurologists. The following variables were recorded: age, sex, and medical history, including hypertension, diabetes, cerebral infarction, coronary heart disease, atrial fibrillation (AF), smoking, and drinking. Time from symptom onset to emergency department presentation (T1) was documented, along with vital signs at admission: temperature, heart rate, breathing, systolic blood pressure (SBP), diastolic blood pressure (DBP), mean arterial pressure (MAP), and blood oxygen saturation (SpO2). Additional parameters included infarction side and variables recorded during ONSD measurement: treatment type (conservative or reperfusion therapy), use of ICP-lowering drugs (including mannitol and hypertonic saline), and mechanical ventilation status. Further data collected included whether DHC was subsequently performed, duration of NICU stay (T2), and laboratory results from the first post-admission blood sample, including white blood cell count (WBC), neutrophil count (N), lymphocyte count (L), and the neutrophil-to-lymphocyte ratio (NLR). GCS and NIHSS scores were recorded at the time of ONSD assessment. Functional outcomes were evaluated using the modified Rankin Scale (mRS) via telephone interview conducted 30 and 90 days after discharge.

### 2.3 ONSD measurement via ultrasound

The initial ONSD measurement (D1) was performed at the bedside in the NICU within 48 h after symptom onset. Subsequent measurements were recorded at 24 h (D2), 48 h (D3), and 96 h (D5) after the initial assessment, whenever clinically feasible. All examinations were conducted by an experienced neurophysiologist to minimize inter-observer variability, using a portable ultrasound device (Qiyou, China) equipped with a linear probe, in accordance with previously established ONSD measurement protocols (15). These standardized methods contributed to the development of the latest ONSD Point-of-Care Ultrasonography Quality Criteria Checklist (ONSD POCUS QCC) (16).

**Figure 1:**
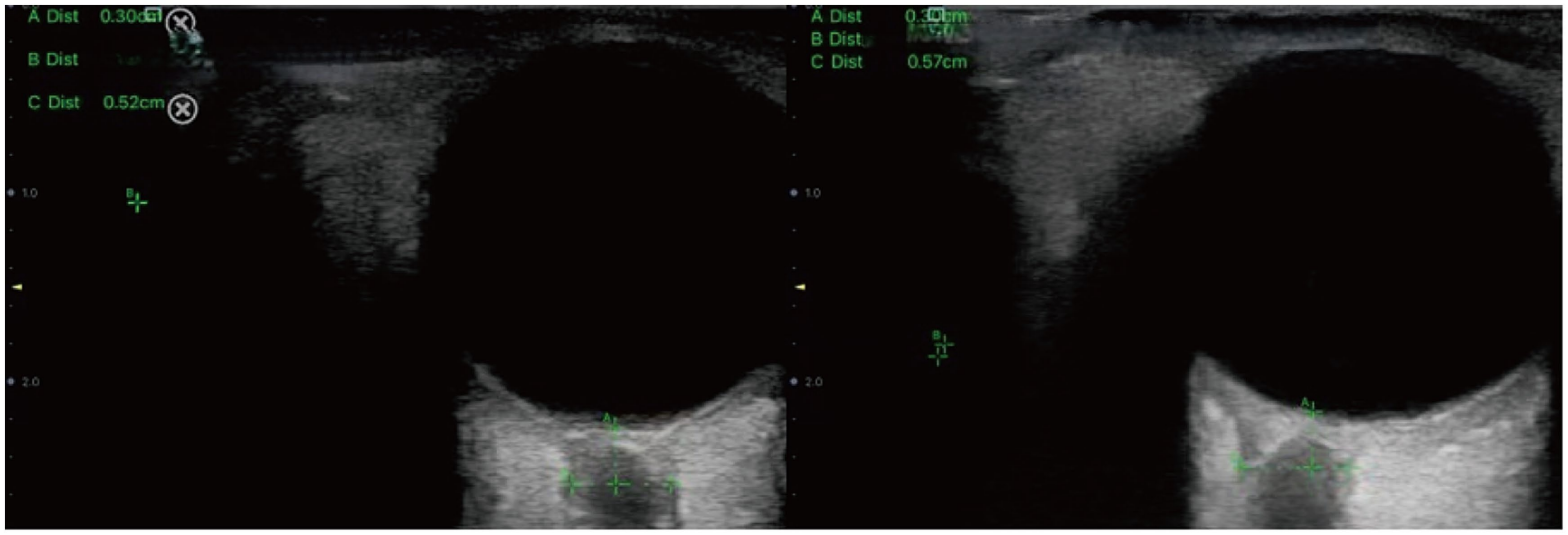
Sonographic measurement of the ONSD 3 mm behind the eyeball. The left ONSD was 0.52 cm in a patient from Group 1, and the right ONSD was 0.57 cm in a patient from Group 2.

During the procedure, patients were positioned supine with their head elevated at 20° to 30°, maintaining a neutral head position and keeping their eyelids naturally closed. A sufficient amount of ultrasound gel was applied to the probe, which was gently placed on the closed upper eyelid. The clearest cross-sectional view of the optic nerve without artifacts was selected for measurement. ONSD was measured 3 mm behind the globe, perpendicular to the optic nerve axis (Figure 1). Three measurements were obtained in both the transverse and sagittal planes for each eye, resulting in six values per eye. The average of these six values for each eye was used for data analysis.

### 2.4 Statistical analysis

Statistical analyses were conducted using IBM SPSS Statistics (version 25), MedCalc (version 23), and PASS 2021 (version 21.0.3). Normally distributed continuous variables were typically expressed as mean ± standard deviation and compared using the independent sample t-test. Non-normally distributed continuous variables are reported as median (interquartile range) and analyzed with the Mann–Whitney U test. Categorical variables were presented as frequency (percentage) and compared using Fisher’s exact test.

Logistic regression analysis was performed on variables that showed significant between-group differences, provided the Events-Per-Variable (EPV) criterion (≥10 events per predictor) was satisfied (17). The initial ONSD measurement, obtained within 48 h after symptom onset, was used for statistical analysis. The association between ONSD and discharge outcomes was evaluated using Point-Biserial or Spearman correlation analysis. Receiver operating characteristic (ROC) curves were used to identify the optimal ONSD cut-off value for predicting clinical deterioration at discharge. Results were reported as area under the curve (AUC), sensitivity, and specificity. A post-hoc power analysis was conducted to assess the reliability of ONSD as a predictive marker. Difference in AUC between ROC curves was compared using the DeLong test. A two-sided *p*-value < 0.05 was considered statistically significant.

## 3 Results

### 3.1 Comparative analysis of demographic and clinical characteristics between the two groups

This study included 35 patients diagnosed with LHI. The median age was 69 (interquartile range: 19.00) years, with 21 males and 14 females. Based on clinical outcomes at discharge, patients were classified into two groups: Group 1 (improved outcome, *n* = 19) and Group 2 (deteriorated outcome, *n* = 16).

Comparative analysis of demographic and clinical characteristics between the two groups identified statistically significant differences in age (*p* = 0.002), female (*p* = 0.002), history of AF (*p* = 0.044), GCS score (*p* = 0.028), affected side ONSD (*p* = 0.002), unaffected side ONSD (*p* = 0.012), and duration of NICU stay (*p* = 0.002) (Table 1). No significant differences were found in other clinical indicators (*p* > 0.05, Supplementary Table 1).

**Table 1:** Difference analysis of demographic and clinical characteristics.

Observation indicators	All Patients (*n* = 35)	Group 1 (*n* = 19)	Group 2 (*n* = 16)	*p* value
Age (years)	69.00 (19.00)	64.00 (21.00)	76.00 (13.00)	0.002
Female (*n*)	14 (40.00%)	3 (15.79%)	11 (68.75%)	0.002
AF	17 (48.57%)	6 (31.58%)	11 (68.75%)	0.044
GCS Score	9.77 ± 3.18	10.84 ± 2.79	8.50 ± 3.23	0.028
ONSD Side (mm)				
Affected	5.50 ± 0.44	5.30 ± 0.42	5.74 ± 0.33	0.002
Unaffected	5.46 ± 0.50	5.27 ± 0.45	5.68 ± 0.46	0.012
T2(days)	6.20(10.80)	12.50(11.80)	4.85(3.58)	0.002

### 3.2 Correlation analysis between ONSD on the affected and unaffected sides and discharge outcomes

**Figure 2:**
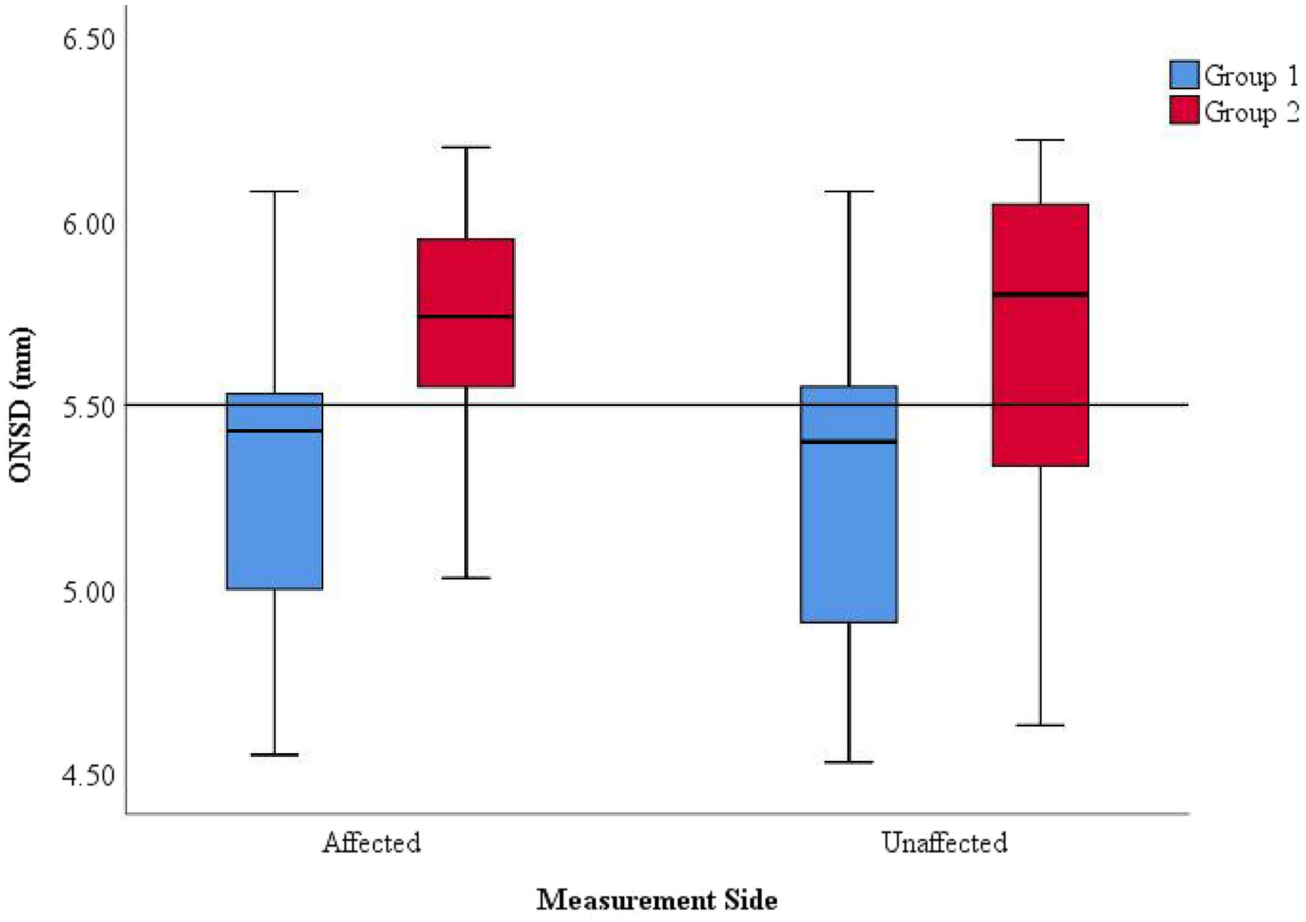
Distribution of ONSD values on the affected and unaffected sides and discharge outcomes. Within the same measurement side, Group 1 (improved outcome) generally exhibited lower ONSD values than Group 2 (deteriorated outcome). Across different measurement sides, the distribution ranges of ONSD values were consistent within each group (Groups 1 and 2).

ONSD values on the affected and unaffected sides were analyzed in the cohort of 35 patients. Subgroup analysis based on discharge outcomes was conducted and presented using box plots (Figure 2).

Within the same measurement side, Group 2 exhibited significantly higher ONSD values compared to Group 1, with measurements frequently exceeding 5.5 mm, suggesting more severe intracranial hypertension (ICH) in Group 2. Among patients with the same discharge outcome, the ONSD on the affected and unaffected sides were relatively consistent, indicating that ONSD measurements from both sides can uniformly reflect the severity of ICP.

A Point-Biserial correlation analysis was conducted between ONSD values on both sides and discharge outcomes in the 35 patients (Supplementary Table 2). The results revealed a significant positive linear correlation between ONSD values and discharge outcomes, indicating that larger ONSD values obtained from any side within 48 h of symptom onset were associated with an increased likelihood of clinical deterioration at discharge.

### 3.3 Predictive performance of ONSD on the affected and unaffected sides for worsened condition at discharge

**Figure 3:**
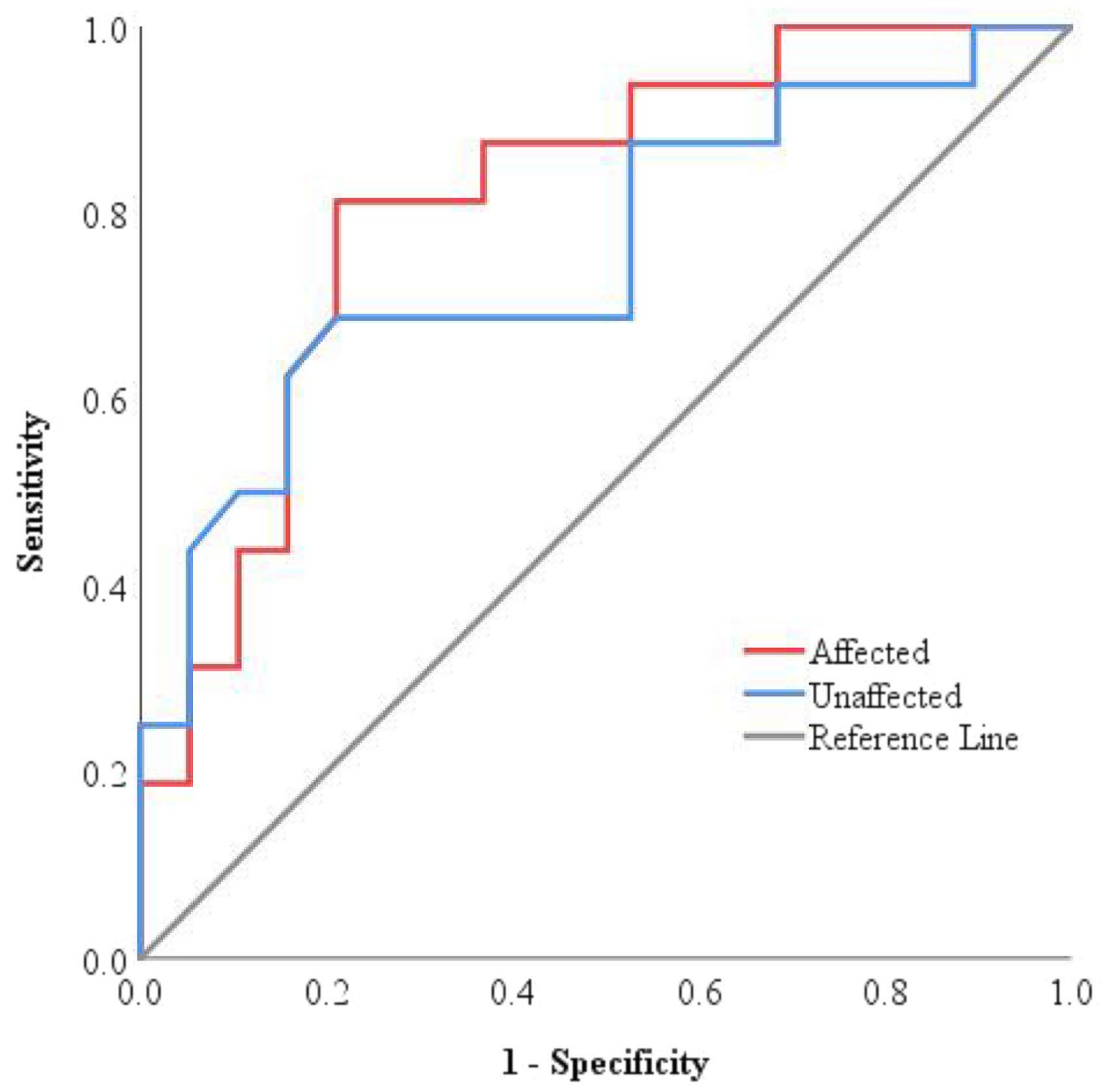
ROC curves for the efficiency of ONSD in predicting worsened condition at discharge. The ROC curves for both sides were located close to the upper-left corner, with an AUC of 0.814 for the affected side and 0.757 for the unaffected side, respectively.

ROC curve analysis was conducted to evaluate the predictive value of ONSD for clinical deterioration at discharge (Figure 3). The results showed that the ROC curves for ONSD measurements for both sides were positioned near the upper-left corner, indicating acceptable predictive performance. The optimal cut-off value of affected side ONSD for predicting deterioration was 5.54 mm, with a sensitivity of 81.3%, a specificity of 78.9%, and an AUC of 0.814, while the corresponding values for unaffected side ONSD were 5.57 mm, 68.8, 78.9%, and 0.757 (Supplementary Table 3), collectively suggesting moderate predictive accuracy. However, the observed width of the confidence interval (CI) suggests that these results should be interpreted with caution.

Since previous studies often used mean bilateral ONSD values as the primary metric, a post-hoc power analysis was conducted to evaluate the predictive reliability of this parameter for discharge outcomes. Based on the observed values (improved group: 5.28 ± 0.43 mm; deteriorated group: 5.71 ± 0.37 mm), the analysis yielded a power of 86.75%, surpassing the conventional threshold of 80%, indicating that the findings are reasonably robust despite the limited sample size.

Comparison of the ROC curves for the affected and unaffected side ONSD, carried out using the DeLong test, revealed no statistically significant difference (AUC difference = 0.058, 95% CI: −0.063 to 0.178, Z = 0.938, *p* = 0.38). This indicates that the predictive performance for discharge outcomes is consistent between the two sides.

### 3.4 Follow-up outcomes of the improved group and the deteriorated group

The mRS was used to evaluate neurological recovery and disability levels at 30 and 90 days after discharge. Follow-up was conducted via telephone interview. Among the 35 participants, 28 patients were successfully followed up, while 7 were lost to follow-up. There was no significant difference in the follow-up rate between the improved group and the deteriorated group (*p* > 0.999, Supplementary Table 4), suggesting that the missing data were likely missing completely at random.

**Figure 4:**
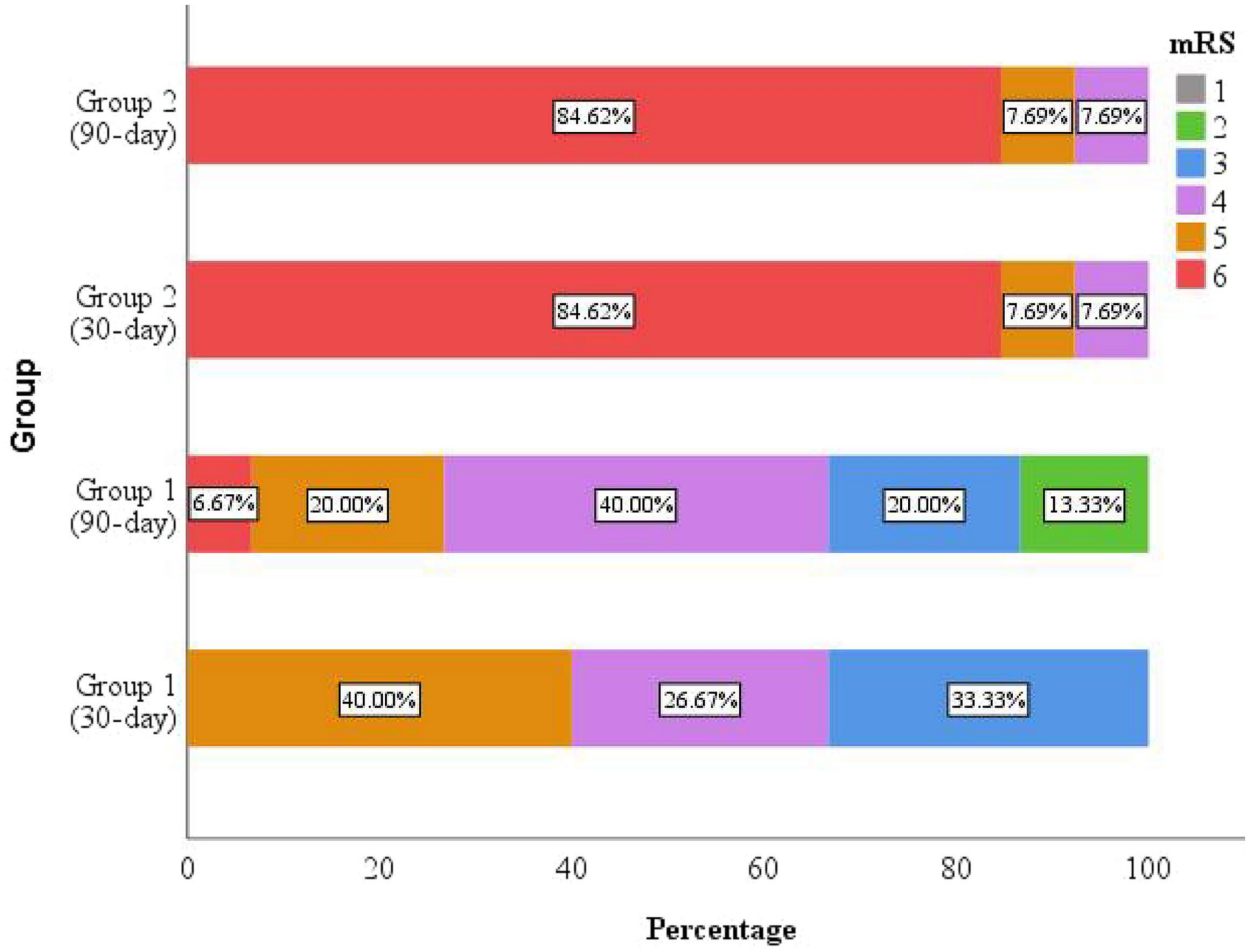
The mRS scores at 30 and 90 days after discharge. Mortality occurred predominantly in the deteriorated group (Group 2), with most deaths occurring within the first 30 days post-discharge. The improved group (Group 1) exhibited clinical improvement during follow-up.

Outcomes of the 28 successfully followed patients were illustrated in a percentage bar chart (Figure 4). The overall 30-day post-discharge mortality was 39.29%, with the majority of deaths occurring in Group 2 (deteriorated outcome). Among survivors, neurological status remained largely unchanged between 30 and 90 days in Group 2, whereas patients in Group 1 (improved outcome) demonstrated significant functional recovery during the same period.

### 3.5 Longitudinal ONSD monitoring

**Figure 5:**
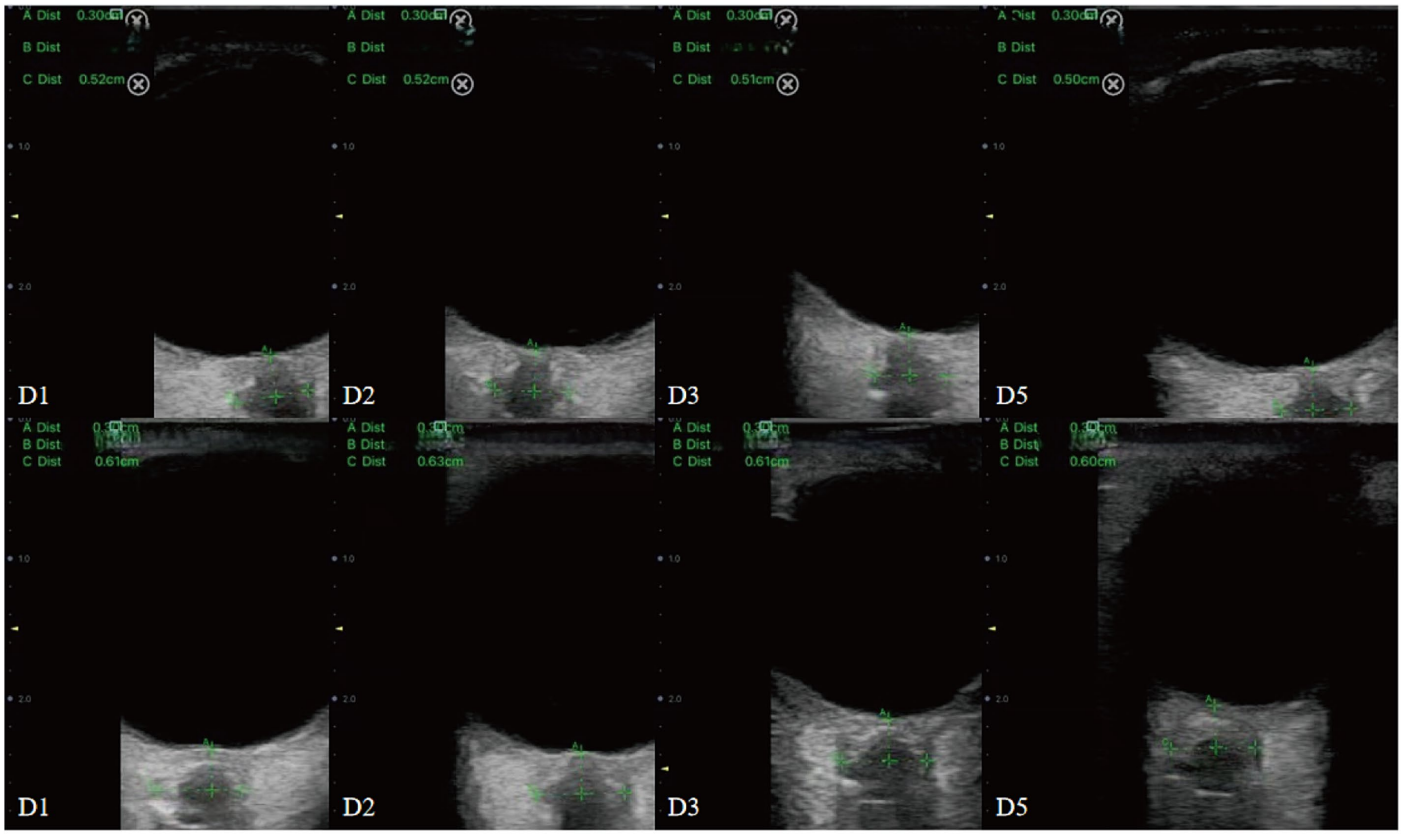
Dynamic changes in ONSD among conservatively managed patients with improved (upper panel) and deteriorated (lower panel) outcomes.

In this study, four consecutive ONSD measurements were obtained from 18 patients with extended NICU stays (Table 2) to characterize longitudinal ONSD changes in the affected and unaffected sides during the early treatment phase for LHI patients (Figure 5). Due to the limited number of patients with repeated measurements and challenges in longitudinal data collection, the analysis was confined to graphical observation of ONSD trends. These preliminary findings are intended to provide a foundational reference for the design of future longitudinal studies on ONSD monitoring.

**Table 2:** Summary of longitudinal ONSD monitoring in patients.

Treatment/Group	Group 1	Group 2	Total
Conservative	4	2	6
Conservative—DHC^*^	2	1	3
Reperfusion	6	0	6
Reperfusion—DHC^*^	0	3	3
Total	12	6	18

* All DHC performed between D1 and D2.

**Figure 6:**
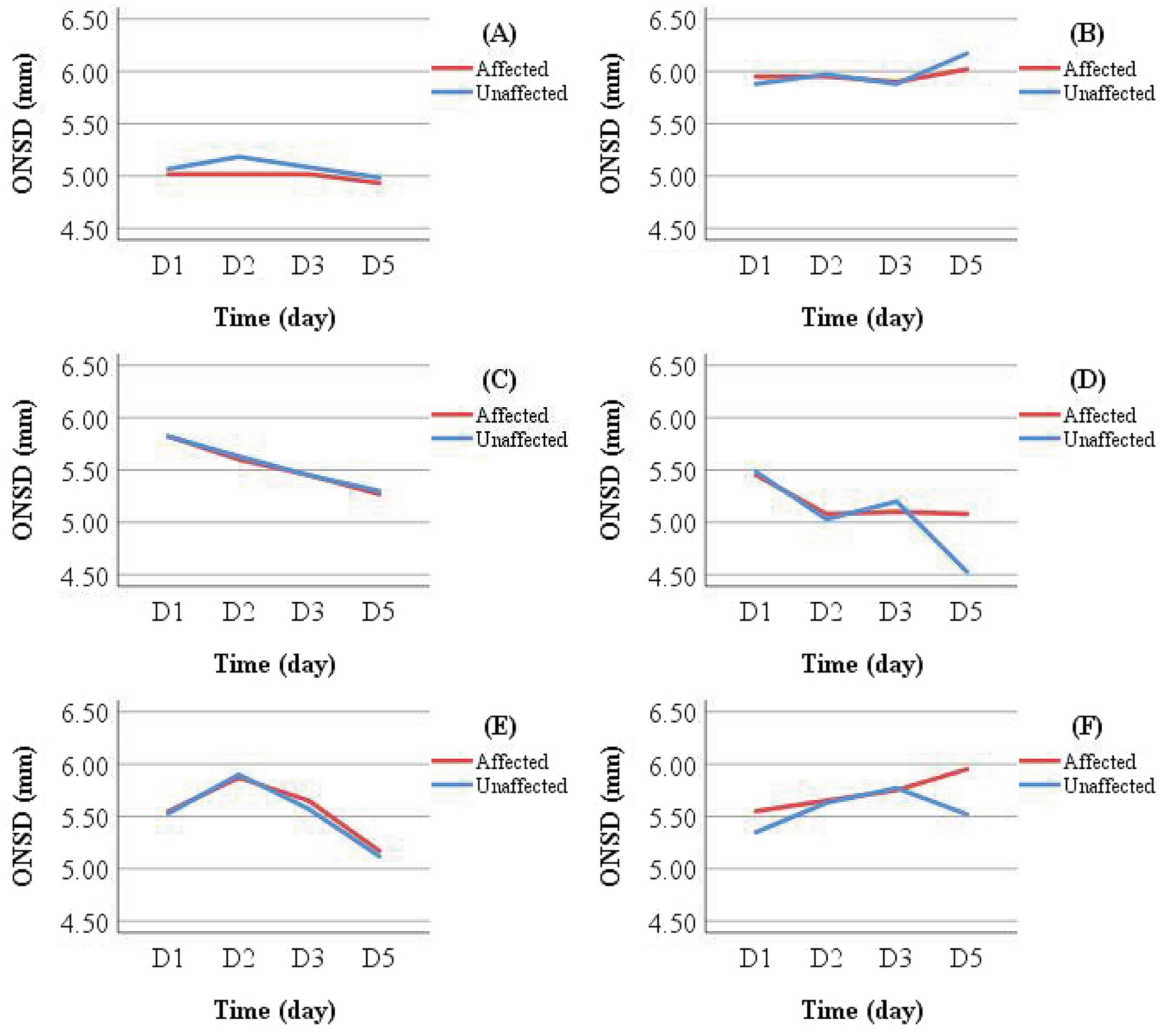
Individual examples of longitudinal ONSD changes in LHI patients. Patients **(A,B)** received conservative treatment; Patients **(C,D)** underwent reperfusion therapy; Patients E and F received DHC (performed between D 1 and D 2) following initial conservative treatment. A decreasing ONSD trend was observed in Patients **(A,C–E)**, who showed clinical improvement at discharge. In contrast, Patients **(B,F)** exhibited an increasing ONSD trend and experienced clinical deterioration.

Line graphs were used to illustrate ONSD changes in six representative patients (Figure 6). Based on discharge outcomes, patients A, C, D, and E were classified into the improved group, while patients B and F were assigned to the deteriorated group. Initial ONSD values exceeded 5 mm in all patients, indicating the presence of ICH within 48 h of symptom onset. Regardless of the treatment administered, the affected- and unaffected-side ONSD curves showed remarkable overlap across all patients. Notably, ONSD values decreased over time in the improved group but increased in the deteriorated group.

**Figure 7:**
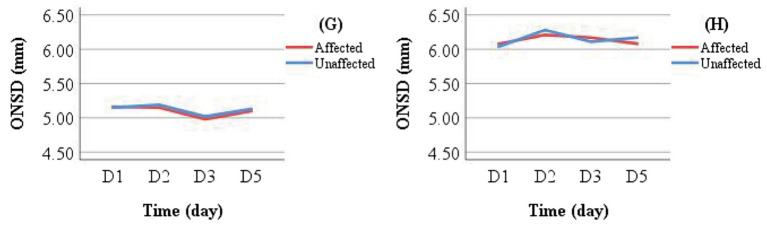
Comparison of ONSD changes between Group G (improved outcome) and Group H (deteriorated outcome) in the conservative treatment cohort.

Patients in the conservative treatment group who did not undergo DHC were further categorized by discharge outcome into Group G (improved outcome) and Group H (deteriorated outcome). Mean ONSD values for the same measurement side at different time points were calculated and visualized using line graphs for intra- and inter-group comparisons (Figure 7). Intra-group analysis revealed overlapping ONSD curves for the affected and unaffected sides. Inter-group comparison showed that ONSD values in the deteriorated group consistently remained above 6.00 mm, whereas those in the improved group stayed below 5.50 mm.

## 4 Discussion

LHI represents a severe form of ischemic stroke, often accompanied by the space-occupying cerebral edema that can lead to midline shift, impaired consciousness, and MCE with potential complications including brain herniation or death (13). Early detection of ICH is therefore critical. Although CT and magnetic resonance imaging (MRI) are the primary imaging techniques for evaluating cerebral infarction severity, transporting unstable LHI patients for these examinations carries risks of secondary injury. Additionally, CT involves radiation exposure and delayed image acquisition, while MRI is contraindicated in patients with metallic implants or claustrophobia. These limitations highlight the need for safer and more efficient monitoring techniques.

Ultrasound-based measurement of the ONSD has gained attention as a non-invasive surrogate for ICP monitoring and has been previously studied in the context of cerebral infarction (9, 18–20). However, most studies have focused on traumatic brain injury, idiopathic intracranial hypertension, and hemorrhagic stroke, with LHI remaining underrepresented due to its relatively low incidence. Existing research on ONSD in LHI has been predominantly retrospective, primarily aimed at predicting malignant progression, yet often lacking standardized definitions of deterioration and documentation of treatments administered before or after ONSD assessment. Furthermore, post-DHC ONSD measurements may not accurately reflect true ICP levels. Importantly, the influence of various treatments and their timing on cerebral edema dynamics and corresponding ONSD values has not been sufficiently examined.

This study employed bedside ultrasonography to measure ONSD and evaluate its predictive value for discharge outcomes in LHI patients. The results demonstrated consistent and moderate predictive performance for both the affected and unaffected sides. This indicates that for patients within 48 h of symptom onset who have received specialized treatment without undergoing DHC, ONSD measurements from either side can effectively predict adverse discharge outcomes. Utilizing a unilateral measurement approach can help reduce procedural time and optimize resource utilization. This technique provides a visual and objective basis for the early detection of ICH, assessment of its severity, prognostic evaluation, and guidance in treatment planning and adjustment. Furthermore, it represents a practical and safe alternative for monitoring and managing ICP in critically ill patients.

Our analysis revealed significantly larger ONSD values in the deteriorated group compared to the improved group. The optimal cut-off values for predicting deterioration were 5.54 mm for the affected side and 5.57 mm for the unaffected side. These values align closely with the cut-off of 5.6 mm reported by Lochner et al. for predicting MMI using ultrasound in 29 patients (9), and 5.52 mm by Guo et al. for predicting malignant edema via CT in 91 LHI patients (10). In contrast, Albert et al. reported a lower cut-off of 5.25 mm for predicting the development of MMI requiring DHC treatment in 38 patients with MCA infarction evaluated by CT (21). These discrepancies may stem from differences in study design, patient demographics, measurement techniques, equipment, and outcome definitions. It is noteworthy that interventions such as ICP-lowering drugs and mechanical ventilation may influence ICP (22) and, consequently, ONSD measurements (23). However, no significant differences were observed in the use of these interventions between our patient groups during ONSD assessment, suggesting that the initial ONSD differences primarily reflected intrinsic ICH rather than treatment effects. Further validation is warranted to confirm these findings.

LHI is marked by acute onset, rapid progression, high rates of disability and mortality, and generally unfavorable prognosis. A meta-analysis confirmed that DHC reduces mortality and improves functional outcomes (1). Although some patients in this study later underwent DHC, it should be noted that certain patients underwent surgical intervention more than 48 h after symptom onset, and were over the age of 60 (13). The impact of surgical timing and patient age on treatment efficacy warrants further investigation.

Additionally, dynamic ONSD monitoring in a subset of patients revealed highly consistent interocular trends before and after treatment, corroborating the findings of Yildiz et al. (12). This supports the potential clinical utility of serial ONSD evaluation. Patel et al., through continuous 2-day ONSD monitoring in acute stroke patients, demonstrated that ONSD was significantly associated with in-hospital mortality and poor prognosis at 6 months; each 0.1 mm increase in ONSD was associated with a 4.2-fold elevated risk of in-hospital mortality in ischemic stroke and a 6.2-fold increase in hemorrhagic stroke (20). These results suggest that longitudinal ONSD monitoring may offer an accessible and objective method for risk stratification in LHI.

The interpretation of our findings should consider several limitations. First, as a single-center study at a tertiary medical hospital, generalizability may be limited, and selection bias is possible, as some patients with severe LHI may not have been hospitalized or may have died before admission. Second, the limited sample size restricted advanced analyses such as subgroup comparisons, multivariate modeling, or assessment of external validity. Future prospective studies should aim to improve sample size estimation and involve multi-center collaborations to enhance robustness. Third, although a single operator performed all ultrasound measurements to minimize inter-observer variability, intra-rater reliability was not formally evaluated. Future work should include assessments of both intra- and inter-rater consistency. Fourth, the absence of invasive ICP monitoring, pre-onset functional scores (e.g., GCS, NIHSS, mRS), and healthy control ONSD measurements limited baseline comparisons and the establishment of reference values. Fifth, due to inconsistent availability of examination results, such as cerebral vascular stenosis and collateral circulation, which are prognostically relevant, these variables could not be systematically analyzed. More standardized protocols are needed in future studies. Sixth, unmeasured confounders, including socioeconomic factors and other unknown variables, may have influenced the results. Seventh, the follow-up was conducted solely by telephone, which may introduce recall and reporting biases. Furthermore, post-discharge events such as complications or rehabilitation were not systematically recorded, potentially confounding the interpretation of long-term outcomes. Subsequent studies should combine clinical visits with telephone interviews to improve data quality.

This study is a single-center investigation with a limited sample size. Future research should adopt large-scale, multi-center designs. The application of artificial intelligence technology may facilitate the identification of risk factors associated with LHI and enable the development of predictive models for quantitative risk assessment. Whether postoperative ONSD measurements accurately reflect ICP in LHI patients undergoing DHC remains uncertain and warrants further investigation. Additionally, the effects of advanced age (over 60 years) and surgical timing (within or beyond 48 h) on clinical outcomes require further elucidation. Ultrasonographic ONSD measurement not only allows for the statistical evaluation of ICP but also supports dynamic monitoring of its variations (24). Although few studies have focused on serial ONSD assessment, its potential clinical utility deserves greater attention in future research.

In conclusion, pre-DHC ultrasound assessment of ONSD within 48 h after symptom onset may help identify LHI patients at risk of deterioration at discharge, with both the affected and unaffected sides showing comparable predictive performance. Several factors, including age, female, history of AF, GCS score, ONSD measurements on either side, and the length of NICU stay, may influence discharge outcomes. The high short-term mortality underscores the urgent need for enhanced post-discharge management. And dynamic ONSD changes exhibit interocular consistency and remain stable before and after treatment interventions, highlighting the potential reliability of serial measurements.

## Data Availability

The raw data supporting the conclusions of this article will be made available by the authors, without undue reservation.

## References

[1] Reinink H Jüttler E Hacke W Hofmeijer J Vicaut E Vahedi K. Surgical decompression for space-occupying hemispheric infarction: a systematic review and individual patient Meta-analysis of randomized clinical trials. JAMA Neurol. (2021) 78:208–16. doi: 10.1001/jamaneurol.2020.3745, PMID: 33044488 PMC7551237

[2] Sun W Li G Liu Z Miao J Yang Z Zhou Q. A nomogram for predicting the in-hospital mortality after large hemispheric infarction. BMC Neurol. (2019) 19:347. doi: 10.1186/s12883-019-1571-4, PMID: 31884967 PMC6935484

[3] Kimberly WT Sheth KN. Approach to severe hemispheric stroke. Neurology. (2011) 76:S50–6. doi: 10.1212/WNL.0b013e31820c35f4, PMID: 21321352

[4] Nag DS Sahu S Swain A Kant S. Intracranial pressure monitoring: gold standard and recent innovations. World J Clin Cases. (2019) 7:1535–53. doi: 10.12998/wjcc.v7.i13.1535, PMID: 31367614 PMC6658373

[5] van der Worp HB Hofmeijer J Jüttler E Lal A Michel P Santalucia P. European stroke organisation (ESO) guidelines on the management of space-occupying brain infarction. Eur Stroke J. (2021) 6:XC–CX. doi: 10.1177/23969873211014112, PMID: 34414308 PMC8370072

[6] Xu N Zhu Q. Optic nerve sheath diameter measured by ultrasonography versus magnetic resonance imaging for diagnosing increased intracranial pressure: a systematic review and meta-analysis. Med Ultrason. (2023) 25:270–8. doi: 10.11152/mu-4037, PMID: 37369031

[7] Berhanu D Ferreira JC Abegão Pinto L Aguiar de Sousa D Lucas Neto L Tavares FJ. The role of optic nerve sheath ultrasonography in increased intracranial pressure: a systematic review and meta analysis. J Neurol Sci. (2023) 454:120853. doi: 10.1016/j.jns.2023.120853, PMID: 37925899

[8] Hansen H-C Lagrèze W Krueger O Helmke K. Dependence of the optic nerve sheath diameter on acutely applied subarachnoidal pressure - an experimental ultrasound study. Acta Ophthalmol. (2011) 89:e528–32. doi: 10.1111/j.1755-3768.2011.02159.x, PMID: 21518306

[9] Lochner P Fassbender K Andrejewski A Behnke S Wagenpfeil G Fousse M. Sonography of optic nerve sheath diameter identifies patients with middle cerebral artery infarction at risk of a malignant course: a pilot prospective observational study. J Neurol. (2020) 267:2713–20. doi: 10.1007/s00415-020-09906-0, PMID: 32440922

[10] Guo Y Chen Y Shen C Fan D Hu X Duan J. Optic nerve sheath diameter and optic nerve sheath diameter/eyeball transverse diameter ratio in prediction of malignant progression in ischemic stroke. Front Neurol. (2022) 13:998389. doi: 10.3389/fneur.2022.998389, PMID: 36158954 PMC9493305

[11] Gao Y Li Q Wu C Liu S Zhang M. Diagnostic and prognostic value of the optic nerve sheath diameter with respect to the intracranial pressure and neurological outcome of patients following hemicraniectomy. BMC Neurol. (2018) 18:199. doi: 10.1186/s12883-018-1202-5, PMID: 30518315 PMC6280512

[12] Yildiz G Acar N Cevik AA Ozdemir AO Metintas S Kaplan D. The evaluation of intracranial pressure evaluation by optic nerve sheath diameter measurement on bedside ultrasonography after ischemic stroke. Clin Neurol Neurosurg. (2021) 209:106914. doi: 10.1016/j.clineuro.2021.106914, PMID: 34507125

[13] Hua X Liu M Wu S. Definition, prediction, prevention and management of patients with severe ischemic stroke and large infarction. Chin Med J. (2023) 136:2912–22. doi: 10.1097/CM9.0000000000002885, PMID: 38030579 PMC10752492

[14] Chinese Society of Neurological Surgery, National Health Commission Stroke Screening and Prevention Engineering Committee, Cross-Strait Medical and Health Exchange Association Neurosurgery Branch Ischemic Cerebrovascular Disease Group. Guidelines for surgical treatment of large hemispheric cerebral infarction. Chin Med J. (2021) 101:3700–11. doi: 10.3760/cma.j.cn112137-20210729-01687

[15] Hirzallah MI Lochner P Hafeez MU Lee AG Krogias C Dongarwar D. Quality assessment of optic nerve sheath diameter ultrasonography: scoping literature review and Delphi protocol. J Neuroimaging. (2022) 32:808–24. doi: 10.1111/jon.13018, PMID: 35711135

[16] Hirzallah MI Lochner P Hafeez MU Lee AG Krogias C Dongarwar D. Optic nerve sheath diameter point-of-care ultrasonography quality criteria checklist: an international consensus statement on optic nerve sheath diameter imaging and measurement. Crit Care Med. (2024) 52:1543–56. doi: 10.1097/CCM.0000000000006345, PMID: 38836697

[17] Peduzzi P Concato J Kemper E Holford TR Feinstein AR. A simulation study of the number of events per variable in logistic regression analysis. J Clin Epidemiol. (1996) 49:1373–9. doi: 10.1016/S0895-4356(96)00236-3, PMID: 8970487

[18] Li C Wang C-C Meng Y Fan J-Y Zhang J Wang L-J. Ultrasonic optic nerve sheath diameter could improve the prognosis of acute ischemic stroke in the intensive care unit. Front Pharmacol. (2022) 13:1077131. doi: 10.3389/fphar.2022.1077131, PMID: 36618944 PMC9816399

[19] Seyedhosseini J Aghili M Vahidi E Shirani F. Association of optic nerve sheath diameter in ocular ultrasound with prognosis in patients presenting with acute stroke symptoms. Turk J Emerg Med. (2019) 19:132–5. doi: 10.1016/j.tjem.2019.07.001, PMID: 31687611 PMC6819719

[20] Patel R Chowdhury MAB Gul S Fahy BG Gonzalez A Fitzpatrick D. Ultrasound of optic nerve sheath diameter and stroke outcomes. Crit Care Explor. (2021) 3:e0565. doi: 10.1097/CCE.0000000000000565, PMID: 34841250 PMC8613366

[21] Albert AF Kirkman MA. Clinical and radiological predictors of malignant middle cerebral artery infarction development and outcomes. J Stroke Cerebrovasc Dis. (2017) 26:2671–9. doi: 10.1016/j.jstrokecerebrovasdis.2017.06.041, PMID: 28736129

[22] Schizodimos T Soulountsi V Iasonidou C Kapravelos N. An overview of management of intracranial hypertension in the intensive care unit. J Anesth. (2020) 34:741–57. doi: 10.1007/s00540-020-02795-7, PMID: 32440802 PMC7241587

[23] Launey Y Nesseler N Le Maguet P Mallédant Y Seguin P. Effect of osmotherapy on optic nerve sheath diameter in patients with increased intracranial pressure. J Neurotrauma. (2014) 31:984–8. doi: 10.1089/neu.2012.2829, PMID: 24372319

[24] Weir CJ Bradford APJ Lees KR. The prognostic value of the components of the Glasgow coma scale following acute stroke. QJM. (2003) 96:67–74. doi: 10.1093/qjmed/hcg008, PMID: 12509651

