# Supplementary material for: Ultrasonographic assessment of optic nerve sheath diameter on the affected and unaffected sides as a predictor of clinical deterioration at discharge in patients with large hemispheric infarction
Source: Front Neurol. 2025 Oct 15;16:1644281. doi: 10.3389/fneur.2025.1644281 (PMC12568332; doi:10.3389/fneur.2025.1644281)
Supplement: Supplementary file 1 [file Table_1.docx]

**Supplementary Table 1.** Difference analysis of demographic and clinical characteristics.

| Observation Indicators | All Patients (n=35) | Group 1 (n=19) | Group 2 (n=16) | *P* Value |
| --- | --- | --- | --- | --- |
| Age (years) | 69.00(19.00) | 64.00(21.00) | 76.00(13.00) | 0.002 |
| Female (n) | 14(40.00%) | 3(15.79%) | 11(68.75%) | 0.002 |
| Medical History (n) |  |  |  |  |
| Hypertension | 21(60.00%) | 12(63.16%) | 9(56.25%) | 0.739 |
| Diabetes | 6(17.14%) | 3(15.79%) | 3(18.75%) | > 0.999 |
| AF | 17(48.57%) | 6(31.58%) | 11(68.75%) | 0.044 |
| Coronary Heart Disease | 3(8.57%) | 2(10.53%) | 1(6.25%) | > 0.999 |
| Cerebral Infarction | 7(20.00%) | 4(21.05%) | 3(18.75%) | > 0.999 |
| Smoking | 10(28.57%) | 6(31.58%) | 4(25.00%) | 0.723 |
| Drinking | 6(17.14%) | 4(21.05%) | 2(12.50%) | 0.666 |
| T1(hours) | 5.00(3.45) | 6.00(5.65) | 4.53(2.41) | 0.337 |
| Temperature (℃) | 36.47 ± 0.19 | 36.52 ± 0.22 | 36.41 ± 0.15 | 0.080 |
| Heart Rate (beats/min) | 91.00(35.00) | 76.00(34.00) | 92.50(33.50) | 0.132 |
| Breathing (breaths/min) | 20.14 ± 3.27 | 20.21 ± 3.52 | 20.06 ± 3.07 | 0.896 |
| SBP (mmHg) | 142.00(35.00) | 141.00(51.00) | 145.50(24.00) | 0.562 |
| DBP (mmHg) | 83.00(14.00) | 80.00(24.00) | 86.00(20.00) | 0.274 |
| MAP (mmHg) | 102.00(9.67) | 100.33(20.67) | 105.33(8.50) | 0.267 |
| SpO_2_ (%) | 98.00(1.00) | 97.00(3.00) | 98.00(1.75) | 0.187 |
| WBC (10^9/L) | 10.13(3.84) | 9.40(3.84) | 10.36(4.15) | 0.573 |
| N (10^9/L) | 8.42(5.07) | 8.42(5.87) | 8.51(4.25) | 0.551 |
| L (10^9/L) | 1.00(0.81) | 1.10(0.89) | 0.98(0.53) | 0.336 |
| NLR | 8.42(5.43) | 7.66(7.28) | 8.72(2.99) | 0.354 |
| GCS Score | 9.77 ± 3.18 | 10.84 ± 2.79 | 8.50 ± 3.23 | 0.028 |
| NIHSS Score | 20.69 ± 4.64 | 19.53 ± 4.02 | 22.06 ± 5.07 | 0.108 |
| Infarcted Side |  |  |  | 0.182 |
| Left | 20(57.14%) | 13(68.42%) | 7(43.75%) |  |
| Right | 15(42.86%) | 6(31.58%) | 9(56.25%) |  |
| ONSD Side (mm) |  |  |  |  |
| Affected | 5.50 ± 0.44 | 5.30 ± 0.42 | 5.74 ± 0.33 | 0.002 |
| Unaffected | 5.46 ± 0.50 | 5.27 ± 0.45 | 5.68 ± 0.46 | 0.012 |
| Treatment Method |  |  |  | > 0.999 |
| Conservative | 15(42.86%) | 8(42.11%) | 7(43.75%) |  |
| Reperfusion  ICP-lowering Drugs  Ventilation | 20(57.14%)  31(88.57%)  11(31.40%) | 11(57.89%)  15(78.95%)  6(31.58%) | 9(56.25%)  16(100.00%)  5(31.25%) | 0.109  > 0.999 |
| Subsequent DHC | 11(31.40%) | 5(26.30%) | 6(37.50%) | 0.716 |
| T2(days) | 6.20(10.80) | 12.50(11.80) | 4.85(3.58) | 0.002 |

**Supplementary Table 2.** Correlation analysis between the ONSD and the discharge outcomes.

| ONSD Side | Correlation Coefficient r | *P* Value |
| --- | --- | --- |
| Affected | 0.510 | 0.002** |
| Unaffected | 0.423 | 0.011* |

* Significantly correlated at the 0.05 level (two-tailed); ** Significantly correlated at the 0.01 level (two-tailed).

**Supplementary Table 3.** Performance of affected- and unaffected-side ONSD in predicting discharge outcomes.

| ONSD Side | AUC | 95%CI | P Valve | Cut-off Value (mm) | Sensitivity  (%) | Specificity  (%) | Youden  index |  |
| --- | --- | --- | --- | --- | --- | --- | --- | --- |
| Affected | | 0.814 | (0.671, 0.957) | 0.002 | 5.54 | 81.3 | 78.9 | 0.602 |
| Unaffected | | 0.757 | (0.591, 0.922) | 0.010 | 5.57 | 68.8 | 78.9 | 0.477 |


**Supplementary Table 4.** Comparison of follow-up outcomes between the improved and deteriorated groups.

| Observation Indicators | All Patients (n=35) | Group 1 (n=19) | Group 2 (n=16) | *P* Value |
| --- | --- | --- | --- | --- |
| Successful Follow-up (n) | 28(80.00%) | 15(78.95%) | 13(81.25%) | > 0.999 |
| 30-day Death (n) | 11(39.29%) | 0(0.00%) | 11(84.62%) |  |
| 90-day Death (n) | 12(42.86%) | 1(6.67%) | 11(84.62%) |  |
| 30-day mRS Score | 5.00(2.00) | 4.00(2.00) | 6.00(0.00) |  |
| 90-day mRS Score | 5.00(2.00) | 4.00(2.00) | 6.00(0.00) |  |
